# Children’s, parents’ and professional stakeholders’ views on power concerning the regulation of online advertising of unhealthy food to young people in the UK: A qualitative study

**DOI:** 10.1371/journal.pone.0268701

**Published:** 2022-06-13

**Authors:** Lauren Carters-White, Shona Hilton, Kathryn Skivington, Stephanie Chambers

**Affiliations:** 1 MRC/CSO Social and Public Health Science Unit, University of Glasgow, Glasgow, United Kingdom; 2 School of Social and Political Sciences, University of Glasgow, Glasgow, United Kingdom; St John’s University, UNITED KINGDOM

## Abstract

Examinations of corporate power have demonstrated the practices and activities Unhealthy Commodity Industries (UCIs) employ to exert their power and influence on the public and health policy. The High in Fat Sugar and Salt (HFSS) product industry have exploited the online environment to market their products to young people. Regulating UCIs’ marketing can limit the power of those industries and is argued to be one of the most appropriate policy responses to such marketing. However, there is minimal consideration of how stakeholders view regulation of online advertising of HFSS products to young people. This UK-focused study addressed this through a secondary analysis of focus groups with young people (n = 15), the primary analysis of focus groups with parents (n = 8), and interviews with professional stakeholders (n = 11). The findings indicated that participants’ views on the regulation of online advertising of HFSS products were informed by how professional stakeholders exerted instrumental, structural and discursive power. Participants cited regulation as a means to re-negotiate problematic power dynamics to increase young people’s and parents’ autonomy over young people’s diets, yet concern remained as to the impact regulation may have on individual autonomy. To garner increased public support for such regulatory policies, it may be beneficial for advocates to emphasise the empowering elements of those regulatory policies. Advocacy actors may wish to shift their framing of regulation from one that focuses on restricting industry practices, to one that centres on empowering individuals.

## Introduction

Over the past decade, public health advocates have emphasised the role unhealthy commodity industries (UCIs) play in driving the increased rates of non-communicable diseases (NCDs), with NCDs contributing to two thirds of deaths and disability worldwide [1]. This has resulted in an increased examination of the role of politics and power in shaping health, and has contributed to the emergence of the field commercial determinants of health (CDoH) [2]. Within public health, there is increasing acknowledgement of the role of corporate power as being central to CDoH, with many practices undertaken by UCIs to exert, maintain and extend their power [3]. In particular, public health advocates and academics have identified the power exerted by the high in fat, sugar and salt [HFSS] product industry as a driver of unhealthy diets through the shaping of food environments and consumer behaviour [1]. For example, a recent analysis of processed food manufacturers’ market strategies reported that they employ a range of techniques to increase and consolidate their power, such as leveraging informational power asymmetries in relations with consumers [4]. Wood et al. [4] reported that processed food manufacturers leverage this informational power through targeting vulnerable population groups with integrated marketing communication practices, or through misleading marketing claims such as employing irrelevant product claims.

Product marketing is another strategy that UCIs employ to exert, maintain and extend their power to drive sales and consumption of their products. Increasingly this marketing is moving to the online environment and policymakers globally are increasingly concerned about this online advertising of HFSS products to young people [5]. This form of online marketing shapes unhealthy dietary preferences amongst young people [6]. It is also a key driver of the broader obesogenic environment [7], ensuring that the HFSS product industry continues to reach new consumers through rapidly developing advertising mediums. In the UK and globally, the public health community have called for such marketing to be regulated [5, 8]. In the UK, the Committee of Advertising Practice, an industry-funded self-regulatory body, regulates the online advertising of HFSS products. The UK Government has recently announced that a ban [with exceptions] of HFSS product advertising online, due to come into force in 2022 [9, 10].

As such, this is an active policy area worthy of academic attention. Despite increased attention on the HFSS product industry’s power as a driver of CDoH and influencer of public health policy [11], few studies have examined various stakeholders views of power within this policy context. This is a critical gap in CDoH research, as the power dynamics that researchers examine are rarely then related to those who they are experienced by. Given that power is a diverse concept, often contentious, and understood in different ways [12], it is even more important that researchers consider it further in this context. This paper reports on a study that examined multiple stakeholders’ [young people, parents, and professional stakeholders] views on the regulation of online HFSS product advertising to young people in the UK, arguing that an examination of power is central to understanding these views on regulation.

### Theoretical framework

Fuchs and Lederer [13] provide a systematic framework for assessing corporate power, specifically business power in global governance. They suggest that to effectively consider the material and ideational sources of power, three distinct forms of power must be considered: 1] instrumental 2] structural, and 3] discursive.

Instrumental power is how corporations seek to influence the political process through practices such as the lobbying of political actors and associated financial activities [13]. By expanding their lobbying practices, corporations can gain a competitive advantage over civil society actors [14, 15].

Structural power concerns the agenda-setting and rule-setting practices that corporate actors employ [3, 13, 16]. Agenda-setting is corporate actors influencing the importance placed on issues, determining what is worthy of policy consideration and what is not [the Overton window [17]]. In addition, corporations can directly influence policy rule-setting, for example through implementing self-regulatory measures [13].

Finally, discursive power “influences the frames of policy problems and solutions, of actors in the political process, and of politics and the political as such” [12, p8]. In particular, discursive power concerns who is considered a legitimate actor within the policy process, as well as contributing to the creation and maintenance of social norms. Although this form of power and agenda-setting within structural power are closely related, discursive power is arguably much more encompassing than agenda-setting alone. It speaks to what is considered socially normal and acceptable, for example in this case the pervasiveness of advertising of HFSS products and society’s normalisation of unhealthy food in society.

Our analysis not only employs this framework to engage with corporate power, as is the focus of Fuchs and Lederer’s [13] work, but extends and develops the framework to include a consideration of non-corporate power [e.g. parents, young people and advocacy groups] as well as State power. In the context of this study, the State refers to government and government bodies. The study demonstrates that analysing corporate power alone [although important] may result in an incomplete understanding of the power dynamics at play within UCI debates [18]. We are unaware of any other analyses of this kind, despite academic attention on the importance of considering power within the obesogenic environment [19, 20].

## Methods

This study used multiple data collection methodologies: 1) secondary analysis of focus groups with young people aged 12–15 years old (n = 15 groups); 2) focus groups with parents of young people aged five to 15 years old (n = 8 groups); and 3) individual semi-structured interviews with professional stakeholders (n = 11).

The University of Glasgow’s (UK) College of Social Sciences Research Ethics Committee provided ethical approval for the studies (application numbers 400160034 and 400150196).

### Data collection

We recruited participants through a variety of means: 1) advertisements distributed through community groups; 2) social media; 3) known contacts in community organisations or professional networks; and 4) snowball sampling. We facilitated focus groups with young people (conducted between June 2016 and July 2016) and parents (conducted between December 2016 and March 2017) in person within community settings or participants’ homes and digitally recorded. We conducted professional stakeholder interviews in person and via the telephone between June 2017 and March 2018, with all digitally recorded. We professionally transcribed the focus groups and interviews checked against the recordings for accuracy.

#### Secondary analysis of focus groups with young people aged 12–15 years old

Research that focuses on young people’s lives is essential for the development of responsive and relevant policies that cater to their needs and concerns [21]. However, the ‘voice’ of young people is often notably absent within the policy process despite being the subject of policy aimed at protecting their interests as a vulnerable group [22, 23]. As such, we deemed it important to consider young people’s views on the regulation of online advertising of HFSS products as it is a policy debate directly relevant to them.

To do so, we undertook a secondary analysis [24] of qualitative data collected for a previous study, which explored young people’s accounts of their experiences of non-broadcast and online advertising of HFSS products. LCW, SC and SH were involved in the original study, and as such have direct and in-depth knowledge of the data.

We invited young people to participate in the study if they were living in Scotland aged between 12 and 15 years of age. In total, 62 young people took part in 15 friendship focus groups (whereby participants were friends before taking part in the study). The smallest group size was three and largest group size seven (the median was four), recruited using a non-probabilistic purposive sampling approach [25] as well as snowball sampling [26]. Twenty-four boys and 38 girls participated from areas with a range of deprivation levels, with nine groups of young people recruited from the least deprived Quintiles (Q4 and 5) as defined by Scottish Index of Multiple Deprivation 16 [27]], five groups from the two most deprived Quintiles (Q1 and 2) and one group from mixed deprivation levels. We obtained consent from both the children who participated in the study, as well as their parents or guardians. Focus groups lasted between 44 minutes and 86 minutes (median of 70 minutes).

#### Focus groups with parents of young people aged five to 15 years old

In policy debates involving children, responsibility varies between governmental responsibility, industry responsibility, and parental responsibility [28]. These debates frame children as vulnerable consumers who parents, particularly mothers, or the State must protect [29, 30].

We included parents living in Scotland with child(ren) aged between 5–15 years of age. We also aimed to recruit participants who lived in a range of socioeconomic areas, and as such targeted recruitment to ensure we achieved this diversity closely as possible.

Thirty parents (29 mothers and one father) living in Scotland participated in eight focus groups (with groups ranging in size from two to five participants, with a median of four per group). We recruited participants across Scotland using a non-probabilistic purposive sampling approach [25] as well as snowball sampling [26]. Participants lived in areas with a range of deprivation levels: 19 of the 30 participants were from the two most deprived Quintiles (Q1 and Q2) in the SIMD16 classification, four were from Quintile 3 (Q3) and four were from the least deprived Quintiles (Q4 & Q5). The age range of their children was 10 months to 29 years, however, all participants had one or more children aged between five and 15 years of age.

Focus groups lasted between 48 minutes and 88 minutes (median of 66 minutes).

#### Individual semi-structured interviews with professional stakeholders

The third set of stakeholders important in this to the debate are those professionals directly involved in the policymaking process (industry, advocacy, government representatives, and academics). Professional stakeholders actively engage in framing to ensure policy meets their interests [31].

We identified participants through their responses to a 2016 consultation by CAP, which was focussed on updating the regulatory code of non-broadcast advertising of food and soft drink to children, including online advertising [32]. The consultation then provided an opportune sampling framework, as each consultee had a ‘stake’ in this debate. Therefore, participants were considered eligible if they were involved in their organisation’s response to the 2016 CAP consultation. We aimed to recruit across a range of industry and non-industry organisations, however recruitment for this section of the study was particularly challenging.

We recruited 11 professional stakeholders across the UK, six were female and five were male. We recruited participants using non-probabilistic purposive sampling approach [25]. Five participants worked within a civil society organisation, two were from the food and beverage industry, one from the advertising industry, two worked in academia and one within a governmental body.

Professional stakeholder interviews lasted between 35 minutes and 69 minutes (median of 51 minutes).

### Data analysis

We employed thematic analysis [33] following Spencer et al.’s [34] analytical hierarchy approach and coded data in NVivo 12. In applying this approach, we generated descriptive accounts identifying and mapping key themes across the data. Next, we generated explanatory accounts to identify patterns of association within the data and consider explanations for why those patterns occur. It was at this point we identified that we required a specific analysis on how participants viewed and responded to power, as it appeared key to understanding their views on regulation of online advertising of HFSS products to young people. During this phase of analysis, we identified themes such as lobbying, management of self-regulation by industry, maintenance of individual autonomy as well as improper State intervention into individual’s lives were being generated through the analysis. A broader concern on power underpinned each of these themes: who has it, who does not have it, and what can be done about problematic power dynamics. We recognised it as being particularly prevalent during discussions surrounding the role of advertising in children’s lives and the regulation of such marketing. As such, we conducted a specific analysis employing Fuchs and Lederer’s [12] framework, and further developed the explanatory accounts generated previously.

We analysed datasets individually at first, to gain an in-depth understanding of the data, before synthesising the three data sets using a thematic approach to generate commonalities and patterns across the data [33] in Microsoft Word.

## Results

The following findings report on how participants’ views of the power dynamics concerning the online advertising of HFSS products appeared to predicate their response to regulation of such an environment. Although the majority of participants appeared to support regulation as a suitable policy option, there was a minority of participants who rejected such regulation. The findings demonstrate, through analysing the various forms of instrumental, structural and discursive power as discussed by the participants, the importance of considering views on power when exploring public health policy options in response to CDoH [18].

Although the focus of the study was online advertising of HFSS products, and participants were reminded of this throughout data collection, participants referred to other forms of advertising to illustrate their points. This may also have been related to the regulatory framework in the UK not only being focused on online advertising, but non-broadcast advertising more generally.

### Instrumental power

There were several instances where participants discussed instrumental power as defined earlier, and this section focuses on how participants responded to the lobbying and financial power of industry and the State. The majority of parents expressed concern over the financial relationship between industry and the State:

*I don’t think that [regulation] would ever come into play because there’s too much a financial gain that there is to be had…the government and the companies are making so much…*

(Parent Focus Group 4 participant 14 –child aged 6)

Parents also expressed concern as to industry’s lobbying practices:

*I mean who else is gonna hold*, *what is essentially a powerful industry with quite*, *the food industry is huge*, *and they have massive access to the law and lobbying MPs*.

(Parent Focus Group 3 participant 10—young people aged four, six and nine)

Non-industry stakeholders expressed a similar discomfort with industry’s investment in lobbying decision-makers. For example, non-industry stakeholders discussed industry’s lobbying of the Department of Culture, Media and Sport (DCMS):

*…we know that behind the scenes there is certainly a lot of lobbying and DCMS and again…why marketing and promotions never happened within the final plan [Childhood Obesity Plan]*.

(Advocacy Stakeholder 3)

In contrast, industry stakeholders considered public health advocacy groups as lobbyists:

*Advertising to young people is a…particularly topical issue at the moment*, *and there’s a lot of interest in the subject*, *particularly from politicians and lobby groups [advocacy groups]*…

(Advertising Industry Stakeholder 5)

This could be considered as industry positioning public health advocacy groups as exerting their own instrumental power.

### Structural power

#### Agenda-setting

Non-industry stakeholders emphasised that online advertising of HFSS products was an issue requiring policy attention, despite the updating of the CAP Code following a public consultation [CAP, 2016]. In addition, non-industry stakeholders highlighted their concern that several points raised by non-industry responders to the consultation were managed incorrectly:

*Limitations*, *I think an awful lot of the concerns that were voiced by the public health community weren’t really dealt with in a way that mirrored the evidence base*.

(Advocacy Stakeholder 2)

Non-industry stakeholders emphasised their organisations’ importance within the policy-making process, underlining the recognition of this by CAP and ASA:

*Through the course of the CAP consultation*, *both before*, *during and after*, *we had meetings with [name] at the CAP and some of his colleagues…even two weeks ago we had a meeting with the ASA…they asked us to come in*, *recognising how…important a stakeholder we now are in this process*.

(Advocacy Stakeholder 3)

In contrast, industry stakeholders attempted to shift attention from the regulation of online advertising of HFSS products to other policy areas they framed as requiring increased focus from public health:

*Not that there’s been…a huge volume of advertising of junk food to young people*, *but if we can say as a sector*, *“Well*, *actually*, *we’ve stopped doing that*, *and we’ve all agreed that we’re not going to do that anymore”*, *and if you take that factor out of the equation*, *then it*, *you know*, *it might help in the longer term to focus efforts elsewhere*.

(Advertising Stakeholder 5)

Industry stakeholders appeared frame further regulation of online advertising of HFSS products as a less desirable policy solution and to close off this policy avenue, in contrast to non-industry stakeholders who viewed as a worthwhile policy solution.

#### Rule-setting

Parents in all but one focus group, a majority of young people, as well as the non-industry stakeholders, argued that the current self-regulatory system was problematic due to industry’s ability to regulate their own advertising practices. As such, these participants expressed support for strengthening the regulatory system and for the State to possess that structural power:

*…it should actually probably be the government because if it was the companies that were like making the product and advertising it they would probably like*…*Yeah just do what they want*.

(Young People’s Focus Group 5 –four males aged 12, 12, 13 and 13)

Conversely, industry stakeholders and parents in one focus group identified the State as possessing excessive structural rule-setting power. Parents in this group expressed frustration with the constraining power of the State:

*Well was that not the whole point of Britain*? *Have we not got freedom*, *d’you know what I mean*? *That’s like the whole point…They’re [the Government] taking away choices*.

(Parent Focus Group 7 participant 25 [child aged seven])

Industry stakeholders expressed a similar concern regarding the State’s rule-setting power:

*…you are always conscious of the threat that if the government chooses to…develop statutory regulation*, *and take over regulation of the industry*, *and that’s clearly not something that we would want*.

(Advertising Industry Stakeholder 5)

Industry stakeholders’ concerns regarding state regulation contrasted with non-industry stakeholders and some parents’ concerns with self-regulation. It appeared that for the industry stakeholders in this study, they aimed to simultaneously reject the rule-setting power of the State, whilst emphasising the success of the industry’s rule-setting:

*…and actually*, *the UK self-regulatory system for advertising is pretty much*, *you know*, *it’s world class*, *and it’s often held up as one of the best examples of a self-regulatory system*.

(Advertising Industry Stakeholder 5)

### Discursive power

#### Legitimate authority figures: The state

Most parents, young people and non-industry stakeholders, considered the State to be a legitimate authority figure. These participants expressed a willingness to place trust in the legitimacy of the State as regulators over industry actors:

*We feel it should be government*. *We definitely*, *we want it regulated on the national level by the government to…create a level playing field*.

(Advisory Stakeholder 1)

In contrast, parents in one focus group expressed resistance to the State as a legitimate authority figure:

*See if they want to advertise Irn Bru [a sugar-sweetened Scottish beverage] or like juice*, *the Lilt…it’s up to the people who want to drink it*. *You can’t shove it [regulation] down people’s throats*.

(Parent Focus Group 7 participant 25 child aged seven)

Industry stakeholders were similarly hesitant towards the State as a legitimate rule-setter, yet they did not state this as explicitly as the participants above. Rather, it seemed they aimed to place doubt over the State’s effectiveness as an authority figure by framing the State as failing to implement a consistent public health strategy to address childhood obesity:

*One of the things that the government looked at when it produced that strategy [Childhood Obesity Plan] was whether or not rules around advertising needed to be tightened*. *We argued…the existing rules were tight enough…when the strategy was published*, *they decided not to pursue any further restrictions*. *And now here we are*, *eighteen months later and…we’re back in having that debate again*.

(Food and Drink Industry Stakeholder 8)

Through the framing of State policy as disjointed yet circular, and that this disjointed process negatively impacted industry, it appeared industry stakeholders attempted to position themselves as ‘targets’ of unsuccessful policymaking, subject to incessant changes out with their control, whilst simultaneously acknowledging their ability to lobby policymakers.

### Legitimate authority figure: Industry

Further to their de-legitimation of the State as an authority figure, industry attempted to legitimise their own role in the policy-making process by emphasising their responsibility:

*And industry’s role is to…be as responsible as possible and not just adhere to the code*, *but always…try and go above it and lean into issues rather than do the minimum*.

(Food and Drink Industry Stakeholder 11)

In contrast to industry participants’ emphasis on their role as legitimate authority figures, most parents, non-industry stakeholders and a minority of young people were sceptical of this legitimacy. Young people provided limited discussion on industry’s role as a legitimate authority figure. Those who did discuss it positioned industry unfavourably:

*And the food industry… … scumbags*.

(Young people’s Focus Group 1 –two females aged 14 and 14 and two males aged 13 and 14)

### Legitimate authority figure: Parents

All participants framed parents as possessing [or should possess] authority over young people’s diets. They framed regulation of online advertising of HFSS as a policy solution, but only if it did not infringe on parental autonomy:

*Yeah…there’s got to be a certain amount of …parents having the confidence and being allowed to parent their own young people as well without the government constantly putting legislation in place ‘oh you’ve got to do this you’ve got to do that’*.

(Parent Focus Group 2 participant 5 young people aged five)

However, what was notable was that some participants believed that increased statutory regulation would provide parents with greater power in this arena:

*So*, *it’s not just about restricting freedom…What we’re saying is let’s take the power and the choice of what your young people are eating*, *let’s take it away from the food industry and give it back to you as parents*.

(Academic Stakeholder 9)

Industry similarly positioned parents as legitimate authority figures. Food and Drink Industry Stakeholder 8 suggested that parents were primarily responsible for young people’s dietary preferences and the impact of online advertising of HFSS products. However, they highlighted industry’s role as being part of the solution, suggesting that industry’s role was to provide help to parents:

*So back to your question about parents you know*, *they—we need to find ways to support them*, *and that isn’t easy*, *it’s clearly not easy*, *‘cause we’re not doing a great job it*, *you know*, *not us*, *not government*, *not schools*.

(Food and Drink Industry Stakeholder 8)

## Discussion

To the best of our knowledge, this study is the first to apply Fuchs and Lederer’s [13] theoretical framework to empirical data about the online environment for HFSS advertising. Participants’ views on power appeared to predicate their acceptability of improved regulation as an appropriate policy response to online advertising. Most participants considered statutory regulation as the most effective means through which to re-shape the power dynamics presented and increase the power of those actors that they deemed as good and legitimate authority figures. However, a small number of participants were resistant towards this form of increased regulation, expressing concern that this would increase the power of the state, which they actively rejected.

Participants viewed lobbying as a key form of instrumental power readily employed by industry actors to influence regulatory debates. As described by Fuchs and Lederer [13], the lobbying of State actors continues to be an important phenomenon seen across a range of industries and global corporations. CDoH literature suggests that UCIs’ lobbying results in weakened public health policy [3, 18], and non-industry stakeholders and some parents echoed this view in our study. These participants suggested that lobbying allowed industry stakeholders to gain privileged access to policy actors [35].

Importantly, most participants [parents and non-industry stakeholders] considered that industry lobbying of State actors ‘omitted’ the public voice from public health debates. This finding suggests frustration that the public voice is not embedded within the policy surrounding the role of regulation or governance in general [36]. Similarly, those who resisted State power appeared frustrated with the current policy status quo, in that it was not conducive to the public’s voice being able to compete with the more prominent voice of the State [36].

The findings suggest that a complex structural power dynamic exists between the State, industry and, to a lesser extent, public health advocacy actors and the public. Both non-industry and industry stakeholders framed each other as a powerful set of actors through their instrumental (lobbying) power within the debate and highlighted that they struggled to compete with the other actors’ power. Although it is well-evidenced that industry actors highlight their importance within the policy process [16, 37–39], citing their industry as critical to remedying the problem, there is less evidence regarding non-industry actors. This is noteworthy as it demonstrates that non-industry actors are employing similar strategies as industry actors, in an aim to ensure their agenda is viewed as legitimate by policymakers and, potentially, the public [40, 41].

Further to their agenda-setting power, participants expressed clear concerns regarding the rule-setting power of industry and the State, with both positioned as possessing too much of this rule-setting power. This tension appeared to arise from a well-established debate between paternalistic policies and ‘nanny-state’ policies and the infringement on individual autonomy [42]. However, the dominant view overall was that industry possessed too much rule-setting power and participants called for the State to conduct the regulation of online advertising of HFSS products to mitigate industry influence. For most participants, industry self-regulation represented a market failure, requiring the State to intervene as the health of the population was at risk [43].

Industry stakeholders, however, were keen to express the success of the current self-regulatory system, in a way that aligns with Otero’s [44] process of ‘neo-regulation’. Neo-regulation suggests that industry does not aim to simply deregulate, but to take control of the regulatory process, ensuring they can shape it to align with their vested interests [18, 41]. In this study, industry stakeholders preferred a continuation of their own regulatory powers, but also wanted to maintain these regulatory powers as ‘evidence’ of their industry responsibility. As such, this study has found that neo-regulation is not only about maintaining control over the regulatory process, but also about positioning industry as a ‘good’ corporate citizen [41, 45].

In terms of discursive power, the findings demonstrated a clear tension in deciding who is and who is not a legitimate actor in the regulation of online advertising of HFSS products to young people. Many participants expressed a desire for parents to possess increased discursive power over young people’s diets, with concern over the legitimacy of industry or the State’s role. Participants’ considered regulation (or lack of) as a means for re-shaping or re-negotiating problematic power dynamics to provide parents, and young people, with increased discursive power and to minimise industry or State control over young people’s diets or policy interventions [36]. Participants who expressed concern that the self-regulatory model inhibited the State’s ability to implement effective regulation aligns with CDoH literature, whereby UCIs have increased their political legitimacy across the globe, reinforcing themselves as authority figures in policy processes [11, 13]. For participants who supported statutory regulation, they framed this form of regulation as an empowering policy, a means to shift power from industry to parents. Whereas participants that considered industry as legitimate authority figures expressed a distrust of the State, and viewed statutory policy as limiting autonomy. The disagreement between participants regarding the role of regulation is one that, on initial reading, may be difficult to address. However, public health ethics literature [46–48] may offer an alternative approach to the framing of regulation of online advertising of HFSS products, or any regulatory policy that may aid in minimising the effects of the CDoH. Dawson [49] proposes that rather than viewing State policies as overly paternalistic, such as regulation of the food industry as infringing on individual autonomy, they perhaps should be viewed as enhancing individual autonomy by minimising industry influence over dietary preferences and norms.

### Policy and practice implications

The key implication of this study is that to garner increased public support for regulatory policies that seek to help parents and young people reclaim young people’s diets, it may be pertinent to emphasise the empowering elements of those regulatory policies that tap into people’s deeply held values or beliefs [40]. As such, advocacy actors may wish to shift their framing of regulation from one that restricts industry practices, to one that empowers individuals as recommended in previous research on public health ethics [49]. One technique may be to employ values-based framing in public health messaging aimed at policymakers and the public, which involves engaging with people’s deeply held values to motivate concern and action, rather than solely focusing on the ‘facts’ of a policy or regulation [40]. This could potentially serve two important purposes. One, it may aid in increasing the likelihood of policymakers implementing policy that seeks to address problematic power dynamics, as previous research indicates the importance of values and stories within the policy making process [40, 50]. Two, it may also aid in increasing public acceptability of such policies.

Furthermore, this study demonstrates the utility of using such distinctions of power, which may help galvanise the public and non-industry actors in addressing the problematic power dynamics explored in this study. By breaking down power into three distinct, albeit inherently intertwined, forms of power we may be better able to develop strategies and policies designed to mitigate some of the problematic effects. Previous studies of power arguably present power as a rather unwieldy, fuzzy concept that makes it difficult to practically see where intervention can be taken, and where increasing public power may occur. This study may be useful in beginning to address this issue, for example aiding families and young people in contributing to the setting of the policy agenda or increasing non-State and non-industry actors’ discursive power.

### Limitations of study

Although this study has presented novel findings, we acknowledge some limitations. Firstly, we employed a secondary analysis of focus groups with young people, and as such the design of that data set was not conducted in line with the focus groups with parents or professional stakeholder interviews. This may have led to a slight incongruence of the data. However, LCW, SH and SC were involved in the original study with young people, and as such brought in-depth knowledge to this current study that otherwise would not have been available. In addition, it is unusual to have a broad range of stakeholders included in a study, particularly those stakeholders who policy is most likely to impact. Furthermore, the larger study [18] did not originally set out to analyse power, and as such questions were not necessarily framed to elicit direct responses on power dynamics. Despite this, the fact that power was so prominent in the data without direct questioning suggests its importance in the debate. It also suggests the importance of conducting further research into the issues of perceptions of power within CDoH when considering the public acceptability of policy designed to address the CDoH. Lastly, the methods we used in the study did not allow for a large number of participants to be included (like a survey may do), however the findings generated from the analysis may be transferable to other policy arenas or unhealthy commodity regulatory debates. To further expand on this research and extend its utility, it may be pertinent to conduct work that explicitly seeks to conduct in-depth analyses on power with the public. This could take place in the form of a population-wide survey, or through a more deliberative process such as citizens’ juries [51].

## Conclusion

The findings demonstrate that in relation to the regulation of online advertising of HFSS products to young people, there is a range of forms of power involved, as evidenced across HFSS product debates internationally [4, 52]. As indicated in Fuchs and Lederer’s [13] framework, these forms of power, particularly for industry actors, can aid in the pursuit of political interests and the exercise of power in policy debates. The findings also demonstrate that although industry power is important, solely focusing on industry power consequently leads to a mischaracterisation of the complex power dynamics at play within a policy debate, particularly when considering the role of regulation as a means of mitigating problematic power dynamics. In addition, this mischaracterisation may lead to a lack of attention paid to what can be done to increase the public’s and non-industry power, as explored above in the Policy & Practice section. Without a clear understanding of the complexity and fluidity of these powers dynamics, we are unable to adequately account for how public health policy interventions may remedy problematic power structures in favour of improving population health.

This study demonstrates the importance of framing of regulation within public health policy debates [40]. To garner increased public support for regulatory policies that seek to help parents and young people gain power over young people’s diets, it may be pertinent to emphasis the empowering elements of those regulatory policies [49] which chime with individuals’ values [40].

## Supporting information

S1 File Parent focus groups. (DOCX)

S2 File Industry interviews. (DOCX)

S3 File Government body interviews. (DOCX)

S4 File CSO interviews. (DOCX)

S5 File Academic interviews. (DOCX)

S6 File Children focus groups. (DOCX)

## References

[1] Wood B, Baker P, Sacks G. Conceptualising the Commercial Determinants of Health Using a Power Lens: A Review and Synthesis of Existing Frameworks. International journal of health policy and management. 2021:-. 10.34172/ijhpm.2021.05 PMC9808328 33619932

[2] Kickbusch I, Allen L, Franz C. The Commercial Determinants of Health. The Lancet Global Health. 2016;4[12]:e895–e6. doi: 10.1016/S2214-109X(16)30217-0 27855860

[3] McKee M, Stuckler D. Revisiting the Corporate and Commercial Determinants of Health. American Journal of Public Health. 2018;108[9]:1167–70. doi: 10.2105/AJPH.2018.304510 30024808 PMC6085040

[4] Wood B, Williams O, Nagarajan V, Sacks G. Market strategies used by processed food manufacturers to increase and consolidate their power: a systematic review and document analysis. Global Health. 2021;17[1]:17. doi: 10.1186/s12992-021-00667-7 33499883 PMC7836045

[5] WHO. Tackling food marketing to children in a digital world: trans-disciplinary perspectives—Children’s rights, evidence of impact, methodological challenges, regulatory options and policy implications for the WHO European Region. 2016.

[6] Coates AE, Hardman CA, Halford JCG, Christiansen P, Boyland EJ. Social Media Influencer Marketing and Children’s Food Intake: A Randomized Trial. Pediatrics. 2019:e20182554. doi: 10.1542/peds.2018-2554 30833297

[7] Swinburn B, Egger G, Raza F. Dissecting Obesogenic Environments: The Development and Application of a Framework for Identifying and Prioritizing Environmental Interventions for Obesity. Preventive Medicine. 1999;29[6]:563–70. doi: 10.1006/pmed.1999.0585 10600438

[8] VicHealth. Values-based messaging for health promotion 2021 [https://www.vichealth.vic.gov.au/media-and-resources/hpcomms

[9] UK Government. Tackling obesity: empowering adults and children to live healthier lives 2020 [https://www.gov.uk/government/publications/tackling-obesity-government-strategy/tackling-obesity-empowering-adults-and-children-to-live-healthier-lives.

[10] UK Government. Consultation outcome: Introducing a total online advertising restriction for products high in fat, sugar and salt [HFSS] 2021 [https://www.gov.uk/government/consultations/total-restriction-of-online-advertising-for-products-high-in-fat-sugar-and-salt-hfss/introducing-a-total-online-advertising-restriction-for-products-high-in-fat-sugar-and-salt-hfss.

[11] McKee M, Steele S, Stuckler D. The Hidden Power of Corporations. BMJ. 2019;364:l4. doi: 10.1136/bmj.l4 30626587

[12] Gaventa J. Finding the Spaces for Change: A Power Analysis. 2006;37[6]:23–33.

[13] Fuchs D, Lederer MML. The Power of Business. Business and Politics. 2007;9[3]:1–17.

[14] Higgott R, Underhill G, Bieler Ae. Non-State Actors and Authority in the Global System. London: Routledge 2000.

[15] Ledgerwood G, Broadhurts AI. Environment, Ethics, and the Corporation. New York: St. Martin’s Press 2000.

[16] Moodie R, Stuckler D, Monteiro C, Sheron N, Neal B, Thamarangsi T, et al. Profits and Pandemics: Prevention of Harmful Effects of Tobacco, Alcohol, and Ultra-Processed Food and Drink Industries. The Lancet. 2013;381[9867]:670–9. 10.1016/S0140-6736(12)62089-3 23410611

[17] Russell NJ. An Introduction to the Overton Window of Political Possibilities. Mackinac Centre for Public Policy 2006.

[18] White LE. Understanding the Policy and Public Debate Surrounding the Regulation of Online Advertising of High in Fat, Sugar and Salt Food and Beverages to Children. Glasgow, Scotland: University of Glasgow; 2020.

[19] Baker P, Gill T, Friel S, Carey G, Kay A. Generating political priority for regulatory interventions targeting obesity prevention: an Australian case study. Social Science & Medicine. 2017;177:141–9. doi: 10.1016/j.socscimed.2017.01.047 28161671

[20] Matheson A. Reducing social inequalities in obesity: complexity and power relationships. Journal of Public Health. 2016;38[4]:826–9. doi: 10.1093/pubmed/fdv197 28158692

[21] Boyden J, Ennew J. Children in Focus: A Manual for Participatory Research with Children Stockholm: Save the Children Sweden; 1997.

[22] Prout A. Children’s participation: control and self-realisation in British late modernity. Children & Society. 2000;14[4]:304–15.

[23] Mehta K, Coveney J, Ward P, Magarey A, Spurrier N, Udell T. Australian children’s views about food advertising on television. Appetite. 2010;55[1]:49–55. doi: 10.1016/j.appet.2010.03.011 20346383

[24] Hinds PS, Vogel RJ, Clarke-Steffen L. The Possibilities and Pitfalls of Doing a Secondary Analysis of a Qualitative Data Set. Qualitative Health Research. 1997;7[3]:408–24.

[25] Guest G, Bunce A, Johnson L. How Many Interviews Are Enough?:An Experiment with Data Saturation and Variability. 2006;18[1]:59–82.

[26] Johnson TP. Snowball Sampling: Introduction. Wiley StatsRef: Statistics Reference Online2014.

[27] Scottish Government. The Scottish Index of Multiple Deprivation 2018 [http://www.gov.scot/Topics/Statistics/SIMD.

[28] Henderson J. Michel Foucault—Governmentality, Health Policy and the Governance of Childhood Obesity. The Palgrave Handbook of Social Theory in Health, Illness and Medicine 2015. p. 324–39.

[29] Colls R, Evans B. Embodying Responsibility: Children’s Health and Supermarket Initiatives. Environment and Planning A: Economy and Space. 2008;40[3]:615–31.

[30] Lister R. Children [but not women] first: New Labour, child welfare and gender. Critical Social Policy. 2006;26[2]:315–35.

[31] Entman RM. Framing: Toward Clarification of a Fractured Paradigm. Journal of Communication. 1993;43[4].

[32] CAP. CAP Consultation: Food and Soft Drink Advertising to Children. UK: Committee of Advertising Practice; 2016.

[33] Floersch J, Longhofer JL, Kranke D, Townsend L. Integrating Thematic, Grounded Theory and Narrative Ananlysis: A Case Study of Adolescent Psychotropic Treatment. Qualitative Social Work. 2010;9[3]:407–25.

[34] Spencer L, Ritchie J, O’Connor W. Analysis: Practices, Prinicples and Processes. In: Ritchie J, Lewis J, editors. Qualitative Research Practice: A Guide for Social Science Students and Researchers. London: Sage Publications Ltd; 2003.

[35] Lacy-Nichols J, Scrinis G, Carey R. The politics of voluntary self-regulation: insights from the development and promotion of the Australian Beverages Council’s Commitment. Public Health Nutrition. 2019:1–12. doi: 10.1017/S1368980019002003 31397246 PMC10200604

[36] Scutchfield FD, Ireson C, Hall L. The Voice of the Public in Public Health Policy and Planning: the Role of Public Judgment. Journal of Public Health Policy. 2004;25[2]:197–205. doi: 10.1057/palgrave.jphp.3190018 15255385

[37] McCambridge J, Mialon MAM, Hawkins BR. Alcohol Industry Involvement in Policy Making. Addiction. 2018:1–35. 10.1111/add.14216 PMC6100095 29542202

[38] Scott C, Hawkins B, Knai C. Food and Beverage Product Reformulation as a Corporate Political Strategy. Social Science & Medicine. 2017;172:37–45. doi: 10.1016/j.socscimed.2016.11.020 27886526

[39] Nixon L, Mejia P, Cheyne A, Wilking C, Dorfman L, Daynard R. "We’re Part of the Solution": Evolution of the Food and Beverage Industry’s Framing of Obesity Concerns Between 2000 and 2012. Am J Public Health. 2015;105[11]:2228–36. doi: 10.2105/AJPH.2015.302819 26378841 PMC4605181

[40] Dorfman L, Wallack L, Woodruff K. More Than a Message: Framing Public Health Advocacy to Change Corporate Practices. Health Education & Behavior. 2005;32[3]:320–36. doi: 10.1177/1090198105275046 15851542

[41] Carters-White L, Chambers S, Skivington K, Hilton S. Whose rights deserve protection? Framing analysis of responses to the 2016 Committee of Advertising Practice consultation on the non-broadcast advertising of foods and soft drinks to children. Food Policy. 2021;104:102139. doi: 10.1016/j.foodpol.2021.102139 34720343 PMC8547229

[42] Capewell S, Lilford R. Are Nanny States Healthier States? BMJ. 2016;355. doi: 10.1136/bmj.i6341 27927622

[43] Calman K. Beyond the ‘Nanny State’: Stewardship and Public Health. Public Health. 2009;123[1]:e6–e10. doi: 10.1016/j.puhe.2008.10.025 19135693 PMC7118790

[44] Otero G. The Neoliberal Diet: Healthy Profits, Unhealthy People. Texas: University of Texas Press; 2018.

[45] Dorfman L, Cheyne A, Friedman LC, Wadud A, Gottlieb. Soda and Tobacco Industry Corporate Social Responsibility Campaigns: How Do They Compare? PLoS Medicine. 2012;9[6]:1–7. doi: 10.1371/journal.pmed.1001241 22723745 PMC3378589

[46] Dawson A. Snakes and Ladders: State Interventions and the Place of Liberty in Public Health Policy. Public Health Ethics. 2016;42:510–3. doi: 10.1136/medethics-2016-103502 27215764

[47] Buchanan DR. Promoting Justice and Autonomy in Public Policies to Reduce the Health Consequences of Obesity. Kennedy Institute of Ethics Journal. 2015;25[4]:395–417. doi: 10.1353/ken.2015.0023 26775879

[48] Beauchamp DE. Public Health as Social Justice. Inquiry. 1976;13[1]:3–14. 130348

[49] Dawson A. Information, Choice and the Ends of Health Promotion. Monash Bioethics Review. 2014;32:106–20. doi: 10.1007/s40592-014-0009-4 25434067

[50] Le Gouais A, Foley L, Ogilvie D, Panter J, Guell C. Sharing believable stories: A qualitative study exploring the relevance of case studies for influencing the creation of healthy environments. Health & Place. 2021;71:102615. doi: 10.1016/j.healthplace.2021.102615 34320460 PMC8520914

[51] McCartney G, Dickie E, Escobar O, Collins C. Health inequalities, fundamental causes and power: towards the practice of good theory. 2021;43[1]:20–39. 10.1111/1467-9566.13181 PMC7894306 33222244

[52] Russell C, Lawrence M, Cullerton K, Baker P. The political construction of public health nutrition problems: a framing analysis of parliamentary debates on junk-food marketing to children in Australia. Public health nutrition. 2020:1–12. 10.1017/S1368980019003628 PMC10200508 31948503

