# Supplementary material for: Children’s, parents’ and professional stakeholders’ views on power concerning the regulation of online advertising of unhealthy food to young people in the UK: A qualitative study
Source: PLoS One. 2022 Jun 13;17(6):e0268701. doi: 10.1371/journal.pone.0268701 (PMC9191734; doi:10.1371/journal.pone.0268701)
Supplement: S1 File — (DOCX) [file pone.0268701.s001.docx]

# Parent Focus Groups: Topic Guide

**Check list – Start**

1. Check everyone received and read the information sheet.
2. Check that consent forms have been signed.
3. Introduce self and research; thank them for their participation.
4. Restate the following:
5. Length of time (approximately 1 hour)
6. Voluntary nature of participation in group discussion
7. Check all participants are happy to be recorded for accuracy
8. Ensure participants understand that they are able to avoid answering questions and may leave discussion at any point
9. Confidentiality – Participants do not mention names of people or discuss the content outside this group (information is kept in locked drawer at MRC/CSO SPHSU and not disclosed to anyone out with the research team)
10. Anonymity – any extracts used in presentations or publications will not use real names (pseudonyms will be used to protect identity so what you say will not be obvious to others)
11. Emphasise group discussion – answers should not be directed to only the facilitator, but to everyone in the group; feel free to ask questions to each other
12. Check if participants have any questions or concerns about the study.
13. Switch on microphone and recorder.
14. Map positions of participants (get postcodes, their age and age of children) and ask participants to introduce themselves.
15. Write out first words of each participant (for later identification).
16. Use topic guide to guide discussion.

**Theme 1: Awareness and understandings of advertising**

- What do you think about the advertising of food and drink to children? (benefits and harms)
- Are you aware of the debates about advertising of food and drinks to children?
- Differences between television and online?

**Theme 2: Online unhealthy food advertising and the home environment (prompts of different online food advertisements examples will be shown to the parents to help generate conversation)**

- Are you aware of your children having seen advertisements of this kind?
- If yes, when and do you remember what kind of advertisement it was?
- Have your children ever asked for a particular product after seeing a food and drink advertisement?
- How do you feel about your children being exposed to online food and drink marketing?
- How do you manage children’s exposure to advertising?
- How do you feel about marketer’s deliberately targeting children with unhealthy food and drink advertisements online?
- Prompt: might need to briefly explain pocket money/pester power theories.

**Theme 3: Regulation and responsibility**

- So you’ve spoken a lot about parent responsibility, what do you think about industry responsibility? What do you think the food industry could do/more of/less of?
- Government responsibility?
- Have you been aware of the debates about regulating the online advertising of unhealthy food and drink to children?
- Who do you think should be responsible for regulation?
- Prompt: government, industry, parents?
- Why?
- Do you feel that your views as parents are represented accurately by groups who claim to represent you?

**Check list – End**

- Is there anything in relation to online advertising of food and drinks to children that we have not spoken about today that you would like to discuss?
- Ensure each person is given a free reply envelope in case they have further information they wish to supply anonymously.
- £15 shopping voucher given and signed for.
- Thank them for their time and ensure they have researcher’s appropriate contact details.
