# Supplementary material for: Children’s, parents’ and professional stakeholders’ views on power concerning the regulation of online advertising of unhealthy food to young people in the UK: A qualitative study
Source: PLoS One. 2022 Jun 13;17(6):e0268701. doi: 10.1371/journal.pone.0268701 (PMC9191734; doi:10.1371/journal.pone.0268701)
Supplement: S2 File — (DOCX) [file pone.0268701.s002.docx]

# Industry Interviews: Topic Guide

| Interviewer: | Date of Interview: |
| --- | --- |
| Interview no: | Organisation/Role: |
| Interview ID: | Descriptor used: |

1. Check Interviewee received and read the information sheet.
2. Check consent form is signed.
3. Introduce self and research; thank Interviewee for agreeing to participate.
4. Restate the following:

- Length of time (approximately 1 hour)
- Voluntary nature of participation
- Check Interviewee is happy to be recorded for accuracy
- Explain that it is okay to avoid answering questions or end discussion at any point
- Confidentiality – emphasis confidentiality rules
- Anonymity – any extracts used in presentations or publications will not use real name (pseudonyms will be used to protect identity so what is said will not be obvious to others)

1. Check for questions or concerns about the study.
2. Switch on microphone and recorder.
3. Ask participant to introduce themselves.
4. Use topic guide themes to guide discussion.

| **Introduction:**  The project examines the views and opinions of both stakeholders and parents in the debate on online advertising of unhealthy food and drink to children. I am interested in providing a clear analysis of the different views in the debate. |
| --- |
| **Theme 1: Stakeholder Position**   - Could you tell me about your involvement in the discussions surrounding the advertising of food and drink to children? (state I am aware of their organisation in the debate but interested to learn more on their views about their involvement) - Why is this issue important to your organisation? (priority?) |
| **Theme 2: Understandings of advertising of unhealthy food and drink**   - What do you think about advertising of food and drink to children? (benefits and harms) - What do you think about the current debate surrounding the regulation of online advertising of unhealthy food and drink to children? - Prompts: example headlines from various media - Where do you think unhealthy food and drink advertising fits into the childhood obesity debate? |
| **Theme 3: CAP consultation and industry regulation**   - Why did your organisation decide to submit to the CAP consultation? - What do you think about the outcome of the CAP consultation? (new measures) - Could you summarise your organisation’s position in the unhealthy online food advertising debate? (acknowledge that you have read policy briefs and statements but this is a changing field) - QUESTION(S) ON INDIVIDUAL RESPONSE - As a member of industry you are liable to current regulations, how do you handle these regulations? (compliance) - IF APPLICABLE: What kind of CSR campaigns do your do and why are these important to your organisation? - What do you think about arguments for increased industry regulation? - Do you think that the new measures will be effective? - What do you think the potential impacts may be on your organisation? - What do you think about other organisations argument that there is sufficient evidence that shows a link between unhealthy advertising to children and their eating habits? - In terms of evidence, where is it that you get your information from? (refer to CAP consultation submission) - Why this source? - Do you value some sources over others? - In several submissions, the UN Convention of the Rights of the Child was used as a reason to defend children against unhealthy food and drink advertising, what do you think about this? |
| **Theme 4: Regulation of unhealthy food and drink advertising to children**   - What do you think about the differences in who regulates television advertising and online advertising? - What would you say the Government’s/industry’s/parents’ role is in regulating the online advertising of unhealthy food and drink to children? - What would you say children’s role is in this? (media literacy in schools) - Parents from a previous part of my study expressed a scepticism surrounding the food industry/government relationship, arguing that they are too imbedded with one another and this has a detrimental impact of improving public health policy – how do you feel about this? (influence on policy) - Who do you think is responsible for regulating the online advertising of unhealthy food and drink to children? - What do you think need to be done to address childhood obesity concerns? |
| **Close of Interview**   - Is there anything in relation to the online advertising of unhealthy food and drink to children that we have not spoken about today that you would like to discuss? - Ensure Interviewee has been given a paid reply envelope in case they want to give further information anonymously. - Thank them for their time and ensure they have appropriate contact information. |
