# Supplementary material for: Children’s, parents’ and professional stakeholders’ views on power concerning the regulation of online advertising of unhealthy food to young people in the UK: A qualitative study
Source: PLoS One. 2022 Jun 13;17(6):e0268701. doi: 10.1371/journal.pone.0268701 (PMC9191734; doi:10.1371/journal.pone.0268701)
Supplement: S4 File — (DOCX) [file pone.0268701.s004.docx]

# Civil Society Organisation: Topic Guide

| Interview ID: | Descriptor used: |
| --- | --- |

1. Check Interviewee received and read the information sheet.
2. Check consent form is signed.
3. Introduce self and research; thank Interviewee for agreeing to participate.
4. Restate the following:

- Length of time (approximately 1 hour)
- Voluntary nature of participation
- Check Interviewee is happy to be recorded for accuracy
- Explain that it is okay to avoid answering questions or end discussion at any point
- Confidentiality – emphasis confidentiality rules
- Anonymity – any extracts used in presentations or publications will not use real name (pseudonyms will be used to protect identity so what is said will not be obvious to others)

1. Check for questions or concerns about the study.
2. Switch on microphone and recorder.
3. Ask participant to introduce themselves.
4. Use topic guide themes to guide discussion.

| **Introduction:**  The project examines the views and opinions of both stakeholders and parents in the debate on online advertising of unhealthy food and drink to children. I am interested in providing a clear analysis of the different views in the debate. |
| --- |
| **Theme 1: Stakeholder Position**  This theme is focused on looking to gain an understanding of your organisation’s position within the debate, as well as your role in the company. This is to give some background knowledge to assist with the rest of the interview.   - Could you tell me about how your work relates to the advertising of food and drink to children? (state I am aware of their organisation in the debate but interested to learn more on their views about their involvement) - Why is this issue important to your organisation? (priority?) |
| **Theme 2: Understandings of advertising of unhealthy food and drink**  This next theme is largely looking to understand your views on food and drink advertising to children, and your opinion on the current reporting and debates surrounding this form of advertising in the childhood obesity discussions.   - What do you think about advertising of food and drink to children? (benefits and harms) - Do you think there is a debate in the policy arena around advertising of food and drink to children? - Why/why not? - Where do you think unhealthy food and drink advertising fits into the childhood obesity debate? |
| **Theme 3: CAP consultation**  This theme is focused on the CAP consultation on the non-broadcast advertising of unhealthy food and drinks to children, as well as your submission to the CAP. It will cover both general questions on the consultation, as well as more focused questions on your organisation’s submission.   - Why did your organisation decide to submit to the CAP consultation? - What do you think about the outcome of the CAP consultation? (new measures) - How do you feel your submission response to the consultation fits into your wider aims? - Do you think that the new measures will be effective? - What do you think the potential impacts may be on your organisation? - What role do you think research evidence has played in influencing the CAP policy on non-broadcast advertising of food and drink to children? - Should it play a more or less prominent role? - What do you think about other organisations argument that there is insufficient evidence that shows a link between unhealthy advertising to children and their eating habits? - In terms of evidence, where is it that you get your information from? (refer to CAP consultation submission) - Why this source? - Do you find some sources of evidence more useful than others? - What evidence benefits your organisation most? - Why did you largely use grey literature in your submission response? |
| **Theme 4: Regulation of unhealthy food and drink advertising to children**  This is the final theme, and it looks more closely at regulating unhealthy online food and drink advertising to children. It covers questions on regulation, responsibility, and data from my previous focus groups conducted with parents.   - What do you think about the difference in who regulates television advertising and online advertising? - What do you think should be the role of…in regulating the online advertising of food and drink to children?  1. Government 2. Industry 3. Parents 4. Children?  - Parents from a previous part of my study expressed a scepticism surrounding the food industry/government relationship, arguing that they are too imbedded with one another and this has a detrimental impact of improving public health policy – how do you feel about this? (influence on policy) - Parents from a previous part of my study expressed that they were unaware that you did this work in this field, and were campaigning for change. How do you feel about this? - What steps do you believe need to be taken to continue to tackle unhealthy food and drink advertising to children? |
| **Close of Interview**   - Is there anything in relation to the online advertising of unhealthy food and drink to children that we have not spoken about today that you would like to discuss? - Ensure Interviewee has been given a paid reply envelope in case they want to give further information anonymously. - Thank them for their time and ensure they have appropriate contact information. |
