# Supplementary material for: Children’s, parents’ and professional stakeholders’ views on power concerning the regulation of online advertising of unhealthy food to young people in the UK: A qualitative study
Source: PLoS One. 2022 Jun 13;17(6):e0268701. doi: 10.1371/journal.pone.0268701 (PMC9191734; doi:10.1371/journal.pone.0268701)
Supplement: S6 File — (DOCX) [file pone.0268701.s006.docx]

# Children’s Focus Groups: Topic Guides

**CHECK LIST- START**

1. Check everyone received and read the information sheet.
2. Check that the consent forms signed. If parent has not signed, then verbal consent must be obtained before interview. Signed consent must be sent for voucher to be issued.
3. Introduce self and research; thank everyone for agreeing to participate.
4. Check if there are any questions or concerns about the study.
5. Restate the following:

- Length of time (approximately just over an hour).
- Voluntary nature of participation in group discussion.
- Provided no objections, keen to record the group discussion for accuracy.
- Fine to avoid answering questions or leave group discussion at any point.
- Confidentiality – Do not mention names of people, do not discuss the content outside this group (information kept in locked drawer in MRC/CSO SPHSU and not disclosed to anyone outside the research team).
- Anonymity - any extracts used in presentations or publications will NOT use your real name. Pseudonyms will be used to protect your identity so what you say will not be obvious to others.
- Emphasise group discussion – answers should not be directed only to facilitator, but to everyone in the group; feel free to ask each other questions.
- Ground rules for group discussion (set by young people) – no phones, let others speak, try not to hold separate conversations as we won’t be able to pick it up.

1. Switch on microphone and recorder.
2. Map positions of participants and ask participants to go around introducing themselves, including finding out young people’s ages. **If any participants are 12 years old, do not show Facebook examples.**
3. Write out first words for each participant – helps later identification
4. Use topic guide themes to guide discussion

**General questions**

When you aren’t at school, how do you spend your time?

Prompt: with friends, watching TV, online

What shows do you tend to watch?

What do you do online?

Prompt: videos, aps, catch up TV.

At home, would your parents want to know how you spend your time online?

[acknowledge families might have different arrangements for pocket money] What would you tend to spend your [pocket] money on when you have it?

Who would say were your favourite celebrities, if any?

Prompt: actors, singers, TV presenters/personalities, sports personalities

**Theme 1: Awareness and understandings of advertising**

Can you think of any adverts that you’ve seen for food and/or soft drinks?

Prompt: for unhealthy and then healthy food.

Can you remember where you saw them?

Prompts: TV, cinema, papers, magazines, billboards/bus stops, social media, [online] games, sporting events etc.

Prompt: can you recall what you liked or disliked about them?

Have you ever bought a product because you saw it advertised somewhere?

Have you asked someone else to buy you a product because you saw it advertised somewhere?

What about others forms of promotion?

Prompt: competitions, special offers, instore packaging? How likely are you to buy something because of that?

What do you think the purpose of advertising is?

How do you think advertising works? [If clarification needed, how does advertising convince people to buy things?]

Who does advertising for food and soft drinks benefit, if anyone?

Who does advertising for food and soft drinks harm, if anyone?

What do you think about advertising of healthy foods?

Have you ever heard anyone talking about food and soft drink advertising before?

Prompt: At school? [Food advertising education is part of the Scottish curriculum]

*Show a short selection of TV advertisements – 4 ads – Skittles, KFC, Maoam, McDonald’s:*

What did you think of these advertisements?

Who do you think ads are aimed to appeal to? Why do you think that?

[If not covered] Do you think ads like these are likely to encourage or discourage you to buy and consume these products? What about other people?

Was there anything else you noticed about the ads that struck you?

*Show children non broadcast ads [Outlined in attached document ‘Non broadcast advertisements’]*

These are some other examples of advertising. In what ways do you think these are different from TV ads?

Prompt: interactive nature, not UK specific?

What do you think of these advertisements?

Who do you think ads are aimed to appeal to? Why do you think that?

Do you think ads like these are likely to encourage or discourage you to buy and consume these products? What about other people?

Was there anything else you noticed about the ads that struck you?

Can you think of anywhere else you’ve seen ads online (or on mobile devices)?

Prompt: apps?

**Theme 2: View about responsibility and regulation**

Have you heard of any rules around food and soft drink advertising here in the UK?

Why might there be rules around this kind of advertising?

Who do you think should make these rules?

Prompt – Government/Parents/Food industry or others?

If you were able to bring in rules, what might you bring in?

Give participants some time to think about it.

**Outline rules around advertising to children:**

*Broadcast advertising* - No advertising unhealthy foods during children’s programming (covers up to 15 years; rules for defining what is a healthy/non healthy food)

Rules on promotions, celebrities, licensed characters

– Ofcom regulates (Government agency).

*Non broadcast advertising* – Food and soft drink advertising targeted at children under 16 must not:

encourage an unhealthy lifestyle [eg snacking rather than eating meals, eating to excess]

Additionally, food and soft drink advertising targeted at children under 12 must not include:

• Celebrities and licensed characters popular with children

• Promotional offers

– Industry fund an independent regulator to monitor this.

What do you think of these rules?

Explain that rules may be changed and outline them, including non government role for non broadcast advertising:

Using characters/celebrities to promote healthy foods

Bans on advertising directed at children in non broadcast media (including online)

Age group that should be considered – under 12s or under 16s?

What do you think of these proposed rules? (prompt whether too focused on TV; ages)

Why might they be good?

Can you think of any problems with these rules?

**CHECK LIST- END**

1. Is there anything about advertising for food and drinks that we have not spoken about today that you would like to discuss?
2. Do you have any questions for me about this study or anything else?
3. Take a note of participant postcodes
4. £15 shopping voucher given and signed for.
